# Interleukin 16 Enhances the Host Susceptibility to Influenza A Virus Infection

**DOI:** 10.3389/fmicb.2021.736449

**Published:** 2021-09-22

**Authors:** Ran Jia, Congwei Jiang, Long Li, Chenxu Huang, Lijuan Lu, Menghua Xu, Jin Xu, Xiaozhen Liang

**Affiliations:** ^1^Department of Clinical Laboratory, Children’s Hospital of Fudan University, Shanghai, China; ^2^Key Laboratory of Molecular Virology & Immunology, Institut Pasteur of Shanghai, University of Chinese Academy of Sciences, Chinese Academy of Sciences, Shanghai, China

**Keywords:** influenza A virus, interleukin 16, interferon, viral replication, lung injury

## Abstract

Influenza A virus (IAV) is a major respiratory pathogen that causes seasonal and pandemic flu, being a threat to global health. Various viral and cellular factors have been characterized to support or limit IAV infection. Interleukin 16 (IL16) has been known as one of the blood signature biomarkers discriminating systemic inflammation due to viral infection vs. other etiologies. Here, we report that the level of IL16 was elevated in the serum samples, lung homogenates, and bronchoalveolar lavage fluid of IAV-infected mice. IL16 overexpression facilitated IAV replication. Conversely, loss of IL16 reduced the host susceptibility to IAV infection *in vitro* and *in vivo*. Furthermore, IL16 deficiency blocked IAV-induced body weight loss and attenuated lung injury in the infected mice. Molecular mechanism analyses further revealed that IL16 could directly inhibit IFN-β transcription and suppress the expression of IFN-β and IFN-stimulated gene. In conclusion, these findings demonstrate that IL16 is a supporting factor for IAV infection.

## Introduction

Influenza A virus (IAV) is a major etiologic pathogen responsible for seasonal flu outbreaks and flu pandemics. IAV is a negative-sense RNA virus and belongs to the orthomyxoviridae family, characterized by its segmented genome which encodes 11 proteins and distinct subtypes based on two surface viral proteins, hemagglutinin (HA), and neuraminidase (NA; Taubenberger and Morens, 2008; Krammer and Palese, 2015). Currently, H1N1 and H3N2 are the two most common circulating influenza A virus subtypes among humans and cause a major public health problem each year (Krammer, 2019). The prevention and treatment of IAV mainly depend on vaccines based on the prevalent IAV strains and antiviral drugs such as oseltamivir and zanamivir. However, unforeseeable antigen drift and antigen shift enable the virus to mutate and develop drug-resistant mutations (Zaraket et al., 2010, 2016; Hurt et al., 2011; Li et al., 2015). This raises the importance of understanding the cellular mechanisms underlying IAV replication and identifying the new host-targeted therapies.

Upon IAV infection, the virion binds to sialic acid receptors on the cell surface membrane and enters the cells through the endocytic pathway (Te Velthuis and Fodor, 2016). After the viral genomes are released into the cytoplasm in the form of viral ribonucleoproteins (vRNPs) consisting of viral RNA and RNA-dependent RNA polymerase, vRNPs are transported into the nucleus and viral RNA undergoes transcription into viral mRNAs which are exported to the cytoplasm, acting as templates to produce new viral proteins (Dou et al., 2018). IAV has eight segments that encode for the 11 viral genes: HA, NA, matrix 1 (M1), matrix 2 (M2), nucleoprotein (NP), non-structural protein 1 (NS1), non-structural protein 2 (NS2), polymerase acidic protein (PA), polymerase basic protein 1 (PB1), polymerase basic protein 2 (PB2), and polymerase basic protein 1–F2 (PB1-F2; Samji, 2009; Eisfeld et al., 2015). After newly translated PB2, PB1, PA, and NP proteins’ entry into the nucleus, genome replication occurs to form progeny vRNPs that are subsequently exported to the cytoplasm with the assistance of the M1 and NS2 proteins and then incorporated into progeny virus particles containing HA, NA, M2, and M1. The new viruses are released from the plasma membrane mediated by two virion surface proteins, M2 and NA (Samji, 2009; Eisfeld et al., 2015).

Influenza A virus interacts with the host factors at every stage of the replication cycle. Numerous cellular proteins have been identified to mediate IAV replication. Among them, multiple proteins are important for supporting IAV replication, such as calcium/calmodulin-dependent protein kinase (CaM kinase) II beta, vacuolar ATPase, and glycogen synthase kinase 3 (GSK3)-beta (Konig et al., 2010). Meanwhile, diverse families of host proteins are implicated as the restriction factors that inhibit IAV replication, such as interferon (IFN)-stimulated genes (ISGs; Sadler and Williams, 2008; Ivashkiv and Donlin, 2014; Iwasaki and Pillai, 2014), tripartite motif proteins (Di Pietro et al., 2013; Fu et al., 2015; Liu et al., 2016; Patil et al., 2018), and DDX21 RNA helicase (Chen et al., 2014). ISG Mx1 could form rings around the ribonucleocapsids of IAV to inhibit viral replication (Gao et al., 2011). ISG15 could inhibit NS1 function (Zhao et al., 2010). Oligoadenylate synthetase-like (OASL) could bind to RIG-I and enhance IFN induction following IAV infection (Villalon-Letelier et al., 2017). Furthermore, type I IFN contributes to the adaptive immune responses against IAV (Curtsinger et al., 2005; Le Bon and Tough, 2008). Hence, the type I IFN signaling is one of the key factors involved in the host defense against IAV.

The previous study has identified and validated a peripheral-blood signature to discriminate host systemic inflammation due to viral infection, and the specific signature genes include ISG15, Interleukin 16 (IL16), OASL, and adhesion G protein-coupled receptor E5 (Sampson et al., 2017). IL16 is first described as a lymphocyte chemoattractant factor in T-lymphoid cells. It is initially translated into a 631 amino acid precursor protein that can be cleaved at residue 511 to generate a 121-residue C-terminal peptide which is subsequently released into supernatant as an aggregate and bioactive form of mature IL16. The mature form of IL16 has been shown to inhibit HIV-1 replication (Baier et al., 1995; Center et al., 1996; Zhou et al., 1997; Zhang et al., 1998). We have recently demonstrated that IL16 regulates gammaherpesvirus pathogenesis by inhibiting viral reactivation (Liu et al., 2020), and IL16 deficiency enhances Th1 and cytotoxic T cell (CTL) response against IAV infection (Jia et al., 2020). In our current study, we showed that IL16 enhanced IAV infection in both human lung epithelial A549 cells and mouse embryonic fibroblasts (MEFs). Furthermore, IL16 deficiency alleviated IAV infection and attenuated the lung pathology in the infected mice.

## Materials and Methods

### Blood Sample Collection

Serum samples from IAV-infected patients (*n*=17) were collected from the Children’s Hospital of Fudan University, Shanghai, China. The inclusion criteria for the patients were as follows: sudden onset of respiratory symptoms, confirmed IAV infection using immunofluorescence assay, no combined infection with other respiratory viruses or bacteria, and no other underlying diseases. Serum samples from age- and gender-matched healthy controls (*n*=16) with no exposure to IAV in 3months were also collected. Serum samples from the mice were obtained from their cheeks on the indicated days after infection. All the serum sample collection was approved by the Ethics Committee of the Children’s Hospital of Fudan University.

### Cell Lines and Plasmids

The Madin–Darby canine kidney (MDCK) cells were kindly provided by Prof. Bing Sun (Institut Pasteur of Shanghai). Wild-type (WT) or IL16-deficient MEFs were isolated from E13.5 embryos as previously described (Liu et al., 2020). A549 cells, MDCK, and MEFs were cultured in Dulbecco’s modified Eagle’s Medium (DMEM; Life Technologies) supplemented with 10% heat-inactivated fetal bovine serum (FBS) and 1% penicillin–streptomycin at 37°C.

The IL16-expressing plasmid with a C-terminal Flag epitope tag was generated by inserting PCR-amplified human IL16 coding fragment into the BamHI and EcoRI sites of a lentivirus vector (pLVX-IRES-Puro; Clontech). The primers used were: forward primer: 5'-CGCGAATTCATGGACTATAGCTTTGATACCACAGCCGAAGAC-3'; reverse primer: 5'-CGGCTCG AGCTACTTGTCATCGTCGTCCTTGTAGTCGGAATCTCCAGCAGCTGTGGTTTCCTTGGACTG-3'. IFN-β-Luc and interferon-sensitive response element (ISRE)-Luc plasmids were kindly provided by Prof. Chen Wang (China Pharmaceutical University).

### Virus, Mice, and Infection

Influenza virus A/Puerto Rico/8/34 (H1N1; PR8) strain was kindly provided by Prof. Haikun Wang, Institut Pasteur of Shanghai (IPS). The PR8 virus was amplified in 10-day-old embryonated hen’s eggs. Briefly, PR8 virus was injected into the allantoic cavity with a 1-ml syringe. Infected eggs were incubated in a 37°C incubator with 82% humidity for 48h and cooled overnight at 4°C. After the allantoic fluid was collected and centrifuged at 2,000rpm for 5min, the supernatants were harvested and filtered through a 0.45μm filter to yield the final virus stock.

Interleukin 16-deficient C57BL/6 mice were generated by Shanghai Model Organisms Center as described previously (Liu et al., 2020). Six- to eight-week-old male WT and IL16 knockout (KO) mice were intranasally inoculated with 8,000 PFU of PR8 viruses in a 20μl volume. All animal experiments were performed in compliance with the guidelines of the Animal Ethics Committee of Institut Pasteur of Shanghai.

### Luciferase Assay and Western Blot

Luciferase assays were carried out as previously described (Tan et al., 2016). IFN-β-Luc or ISRE-Luc plasmids were transfected into A549 cells together with the Renilla reporter plasmid as an internal control in the presence or absence of IL16-Flag expressing plasmids using the Neofect transfection reagent (Gene delivery biosystems). Luciferase assays were carried out in triplicate. Luciferase activity was measured using the Dual Luciferase Reporter Gene Assay kit (Beyotime Biotechnology) according to the manufacturer’s instructions and normalized to Renilla activity.

Western blot was performed using the specific antibodies. NP antibody was kindly provided by Prof. Bing Sun, IPS; NS antibody was kindly provided by Prof. Dimitri Lavillette, IPS; HA antibody (PA5-34929) was from Invitrogen; HRP-conjugated Flag antibody (A8592) was from Sigma; IL16 antibody (abs136120) was from Absin Bioscience; GAPDH antibody (sc-32233) was from Santa Cruz Biotechnology.

### Quantitative RT-PCR

Total RNA was extracted using TRIzol reagent (Life Technologies) following the manufacturer’s protocol, followed by cDNA synthesis with ReverTra Ace qPCR RT Master Mix (TOYOBO) according to the manufacturer’s instructions. Quantitative PCR was performed using KOD SYBR® qPCR Mix (TOYOBO) on the Light Cycler 96 instrument (Roche). Relative mRNA levels were calculated by applying the 2^−ΔΔ*C*t^ method using GAPDH as a reference housekeeping gene. In our study, the control sample usually means uninfected sample. The fold increase was relative to the mRNA level of IL16-overexpressing sample at 0h post-infection. The primers used were: NA: 5'-TGTTGA TGGAGCAA ACGGAGTA-3' and 5'-CTCAAACCCAT GTCTGGAACTG-3'; HA: 5'-GGCCCAACCA CAACACAAAC-3' and 5'-AGCCCTCCTTC TCCGTCAGC-3'; M: 5'-GACCRA TCCTG TCACCTCTGAC-3' and 5'-AGGGCATTYTGGACAAAKCGTCTA-3'; mIFN-β: 5'-AGCTCCA AGAA AGGACGAACAT-3' and 5'-GCCCTGTAGGTGAGGTTGATCT-3'; mISG15: 5'-CAGGACGGTCTTACCCTTTCC-3' and 5'-AGGCTCGCTGC AGTTCTGTAC-3'; mGAPDH: 5'-AACGACCCCTTCAT TGACCT-3' and 5'-ATGTTAGTGGGGTCTCGCTC-3'; hIFN-β: 5'-GTCAGA GTGGAAATCCTAAG-3' and 5'-ACAGCATCTGCTGGTTGAAG-3'; hISG15: 5'-AGATCAC CCAGAAGATCGGC-3' and 5'-GAGGTTCGTCGC ATTTGTCC-3'; hGAPDH: 5'-AGAAGG CTGGGGCTCATTTG-3' and 5'-AGGGGCCATCCACAGTCTTC-3'.

### Virus-Binding Assay

Madin–Darby canine kidney cells transfected with vector or IL16-expressing plasmids were detached using Trypsin. The detached cells were incubated at 37°C in DMEM supplemented with 10% FBS for 30min, followed by washing with pre-chilled serum-free DMEM twice. The cells were then incubated with the pre-chilled PR8 virus at an MOI of 10 at 4°C for 30min with rotation. After washing with ice-cold PBS, the cells were immediately fixed with 4% paraformaldehyde and probed with anti-HA rabbit monoclonal antibody (PA5-34929; Invitrogen) for 1h on ice, followed by incubation with donkey anti-rabbit IgG (H+L) Alexa Fluor 488-conjugated secondary antibody (A21206; Invitrogen) for 30min. The cells were subsequently subjected to flow cytometry analysis.

### Virus Entry Assay

A549 monolayer cells were washed twice with pre-cooled serum-free DMEM and inoculated with PR8 virus (MOI=1) for 30min at 4°C. Cells were then washed twice with pre-cooled serum-free DMEM and added with DMEM supplemented with 10% FBS and 1% penicillin-streptomycin, followed by incubation at 37°C. The infected cells were collected at the indicated time points and virus entry into the cells was quantified by qRT-PCR with the specific primers corresponding to PR8 HA and M genes.

### Influenza A Virus Titering

Viral titer was measured by plaque assay in MDCK cells. Briefly, MDCK cells were seeded in a 96-well plate and washed twice with serum-free DMEM before infection. After a series of tenfold dilutions of lung homogenates or culture supernatants were added to MDCK cells and incubated for 30min at 37°C, the cells were washed twice and added with semisolid culture medium [1×DMEM, 0.75% methylcellulose, 1μg/ml Tosyl phenylalanyl chloromethyl ketone (TPCK)-treated trypsin (Sigma), 1% penicillin-streptomycin]. After 24h, cells were fixed with 4% paraformaldehyde for 5h at room temperature (RT) and the semisolid medium was subsequently removed. Cells were incubated with rabbit-anti-NP antibodies overnight, followed by incubation with HRP-conjugated-goat-anti-rabbit IgG (R2004; Sigma) for 2h and stained with tetramethylbenzidine substrate (MABTECH) for 15min. The plaques were counted under the microscope. The virus titer was displayed as PFU/ml and determined by dilution factors and plaque numbers.

### Bronchoalveolar Lavage Fluid and Lung Homogenates Preparation

Bronchoalveolar lavage fluid (BALF) was acquired using a disposable syringe needle with an outer meter of 1.2mm. Briefly, the mouse was anesthetized and the trachea was exposed. A small semi-excision of the trachea was made by a scissor to allow the needle to pass into the trachea. Five hundred microliter of sterile PBS was injected into the lungs using a 1-ml syringe and aspirated back. Repeat the injection and aspiration for two times to get the final BALF samples.

Lungs were harvested and homogenized in DMEM using an automatic tissue grinder (Shanghai Jingxin Co., Ltd) at 60Hz for 1min. The homogenates were clarified by centrifugation at 8,000rpm for 10min. For measuring the virus titers in the lungs of infected mice, the supernatants of homogenates were subjected to quantify the infectious viruses on MDCK cells. For IL16 detection by ELISA and immunoblot analyses, the supernatants were diluted appropriately before the detection.

### ELISA Assay

IL16 levels were detected using a Quantikine ELISA kit for human IL16 (D1600; R&D Systems) and a DuoSet ELISA kit for mouse IL16 (DY1727; R&D Systems). IFN-β levels were measured using a VeriKine mouse IFN-β ELISA Kit (42400; PBL assay science). Briefly, the samples and the standards were added to the wells pre-coated with capture antibody and incubated for 2h at RT, followed by a total of three washes. After incubation with the detection antibody for another 2h at RT, the substrate solution was added and the optical density was determined at a wavelength of 450nm.

### Flow Cytometry Analysis

Lung single cells were prepared as previously described (Longhi et al., 2009). Briefly, lungs were digested in RPMI 1640 supplemented with 20μg/L Liberase (Roche) and 25μg/L DNase I (Roche) at 37°C for 30min. After red blood cells were removed using the ammonium-chloride-potassium buffer, lung single cells were blocked using anti-CD16/32 (553142, BD Biosciences) and stained with BV510-anti-CD11b (562950, BD Biosciences), APC-anti-CD11c (17-0114-81, eBiosciences), PE-anti-F4/80 (565410, BD Biosciences), BV650-anti-Ly6G (740554, BD Biosciences), and PE-Cy7-anti-Ly6C (4341610, Invitrogen). The cells were subsequently fixed and permeablized using BD CytoFix/CytoPerm Kit (BD Biosciences) according to the manufacturer’s instructions. Intracellular virus staining was performed using an anti-IAV M2 antibody (Abcam) and a FITC conjugation kit (Abcam). Flow cytometry was performed using BD LSRFortessa (BD Biosciences) and raw data were analyzed using FlowJo software.

### Histopathological Analysis

The tracheas of euthanized mice were cannulated and the lungs were inflated with 500μl of PBS. The inflated lungs were isolated from mice and submerged in 4% formalin for at least 10h. After fixation, the lungs were embedded in paraffin and cut into 5μM sections. The lung sections were stained with hematoxylin–eosin (H&E; Servicebio). The images of lung sections were captured by a microscope (Leica biosystems) at resolutions of 100× and 400× magnification.

### Statistical Analysis

Statistical analysis was performed using Prism (GraphPad Software). The data are presented as mean±SD and differences between groups of research subjects were analyzed for statistical significance with two-tailed unpaired *t*-tests, except that the serum IL16 level is presented as median±interquartile range and statistically analyzed by Mann–Whitney *U* test. A value of *p*<0.05 was considered significant.

## Results

### The Level of IL16 Is Increased in IAV-Infected Children and Mice

**Figure 1:**
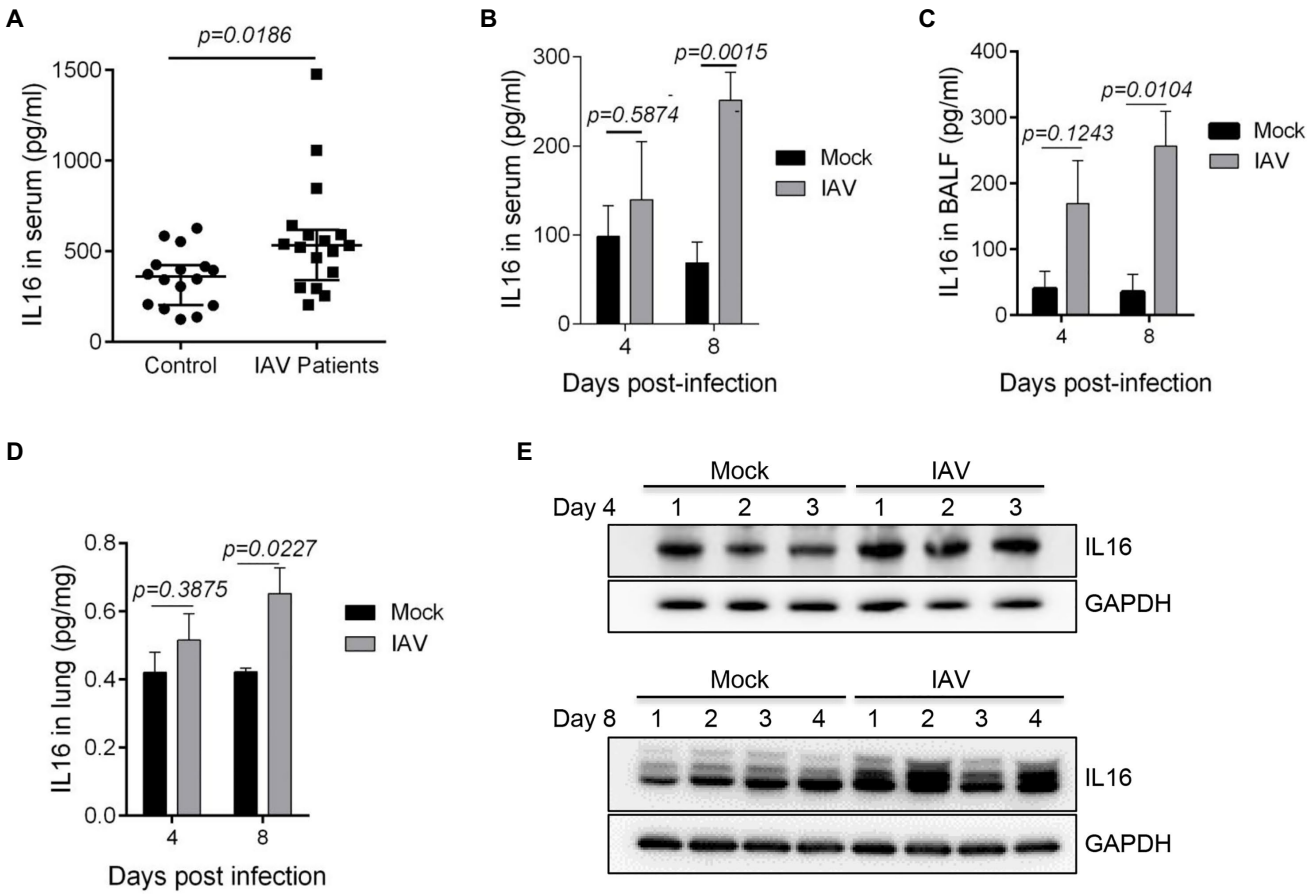
The level of interleukin 16 (IL16) is increased in influenza A virus (IAV)-infected children and mice. **(A)** Sera from IAV-infected children (*n*=17) and healthy controls (*n*=18) were detected for IL16 by ELISA. **(B–E)** C57BL/6 WT mice (6–8weeks) were infected with mock (*n*=5) or PR8 virus (8,000 PFU/mouse, *n*=6). ELISA was performed to detect the level of IL16 in serum samples **(B)**, bronchoalveolar lavage fluid (BALF; **C**), and lung homogenates **(D)** from IAV-infected mice and control mice at day 4 and day 8 post-infection. The lung homogenates were also subjected to immunoblot analyses for IL16 detection on day 4 and day 8 post-infection, number 1–4 represents individual mouse **(E)**. A value of *p*<0.05 was considered significant.

Since IL16 expression is a biomarker signature to systemic inflammation due to virus infection including IAV (Sampson et al., 2017), we detected the IL16 level in the serum samples from IAV-infected children. We observed a statistically significant increase in the serum IL16 level of IAV-infected children, although the discrepancy was minor (Figure 1A). Similarly, IAV PR8-infected mice also showed a significant increase in IL16 production in serum samples (Figure 1B), BALF samples (Figure 1C), and lung homogenates (Figures 1D,E), indicating that IL16 is elevated during IAV infection in both humans and mice.

### IL16 Overexpression Facilitates IAV Replication

**Figure 2:**
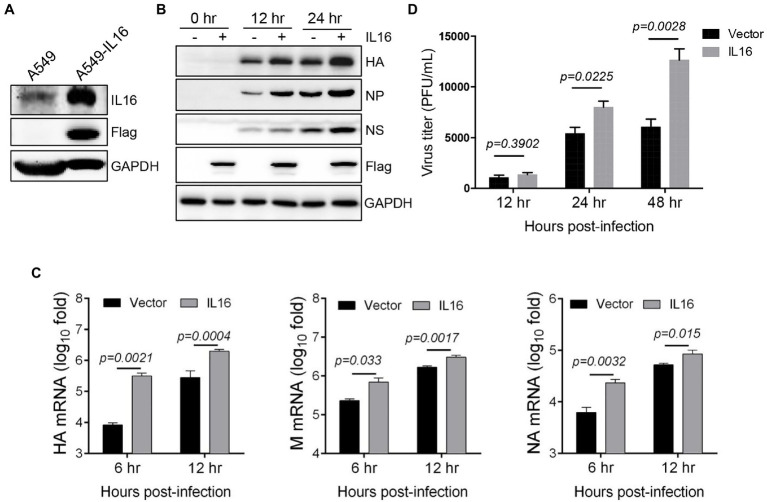
IL16 overexpression promotes IAV replication. **(A)** Detection of endogenous IL16 expression by immunoblot analyses. A549 cells transfected with IL16-Flag expressing plasmids was used as a positive control. GAPDH was used as a loading control. A549 cells were transfected with vector or IL16-Flag-expressing plasmid, followed by infection with PR8 virus (MOI=0.1) for indicated time points. **(B)** The infected cells were harvested at 0, 12, and 24h post-infection and subjected to immunoblot analyses with the indicated antibodies. GAPDH was used as a loading control. **(C)** The infected cells were harvested at 6 and 12h post-infection. The relative RNA levels of viral hemagglutinin (HA), M, and neuraminidase (NA) genes were measured by quantitative RT-PCR (qRT-PCR). Histograms represent mean±SD of triplicate samples (two experiments). A value of *p*<0.05 was considered significant. **(D)** The supernatant was collected from the infected cells at 12, 24, and 48h post-infection, and virus titer was measured by plaque assays. Histograms represent mean±SD of triplicate samples (two experiments). A value of *p*<0.05 was considered significant.

Given that IAV infection stimulates the production of IL16, we wonder whether IL16 plays any role in IAV infection. To test this, we used human lung epithelial cells (A549) which expressed a low level of endogenous IL16 (Figure 2A). A549 cells were transfected with IL16-Flag-expressing plasmid or vector alone for 24h, followed by infection with PR8 virus at an MOI of 0.1. IL16 overexpression upregulated the protein level of HA, NP, and NS proteins of the PR8 virus (Figure 2B). The qRT-PCR analyses showed that the mRNA levels of PR8 HA, M, and NA were significantly higher in the cells transfected with IL16 as compared to the cells transfected with vector alone (Figure 2C). Consistently, ectopic IL16 expression increased PR8 virus production, as determined by a plaque assay (Figure 2D). These data suggest that IL16 promotes IAV infection.

**Figure 3:**
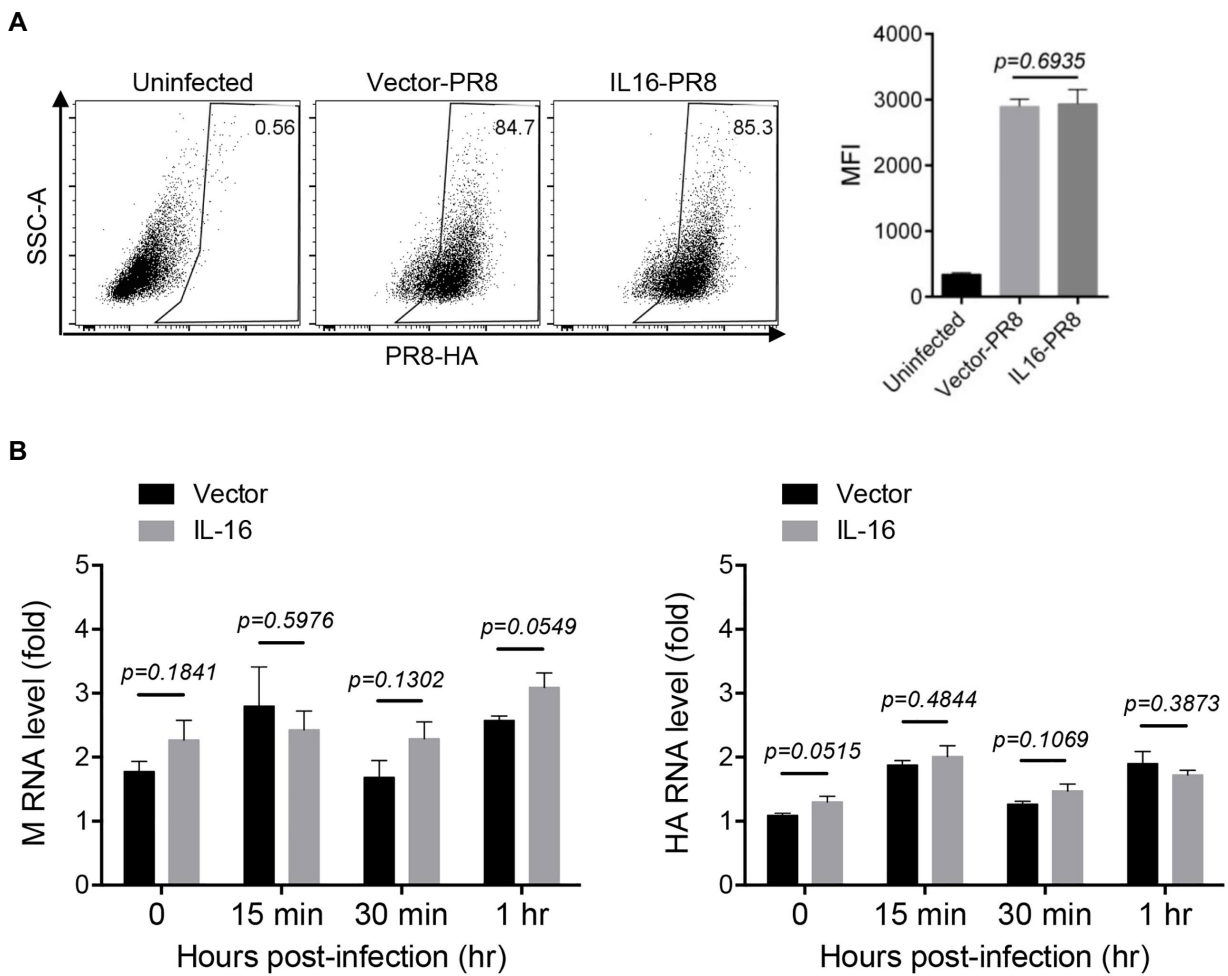
IL16 overexpression does not affect IAV entry. **(A)** Madin–Darby canine kidney (MDCK) cells were transfected with vector or IL16-expressing plasmid for 24h, followed by infection with PR8 virus (MOI=10) at 4°C. The infected cells were surface-stained for HA and subjected to flow cytometry analyses. **(B)** A549 cells were transfected with vector or IL16-expressing plasmid for 24h and were infected with PR8 virus (MOI=1) at 4°C, followed by incubation at 37°C for 15min, 30min, and 1h. The infected cells were harvested and subjected to qRT-PCR analyses with M and HA specific primers. Histograms represent mean±SD of triplicate samples (two experiments). MFI represents mean fluorescent intensity. A value of *p*<0.05 was considered significant.

Next, we determined whether IL16 had any effect on IAV entry. To see the effect of IL16 on virus binding, MDCK cells were transfected with IL16-expressing plasmid or vector alone, followed by incubation with PR8 virus (MOI=10) at 4°C for 30min. The infected cells were then surface-stained for PR8 HA and subjected to flow cytometry analyses. The levels of PR8 virus binding to MDCKs were similar regardless of IL16 overexpression (Figure 3A). For entry assay, A549 cells were transfected with IL16-expressing plasmid or vector and incubated with PR8 virus (MOI=1) at 4°C for 30min. The infected cells were subsequently moved to 37°C and incubated for 0, 15min, and 30min, and 1h. The virus entry was measured by the M and HA RNA levels of the PR8 virus through qRT-PCR analyses. There was no significant difference for M and HA RNA levels between IL16-expressing cells and control cells after PR8 infection for 15min, 30min, and 1h (Figure 3B), suggesting that IL16 does not affect the entry-level of PR8 virus.

### IL16 Deficiency Inhibits IAV Replication

**Figure 4:**
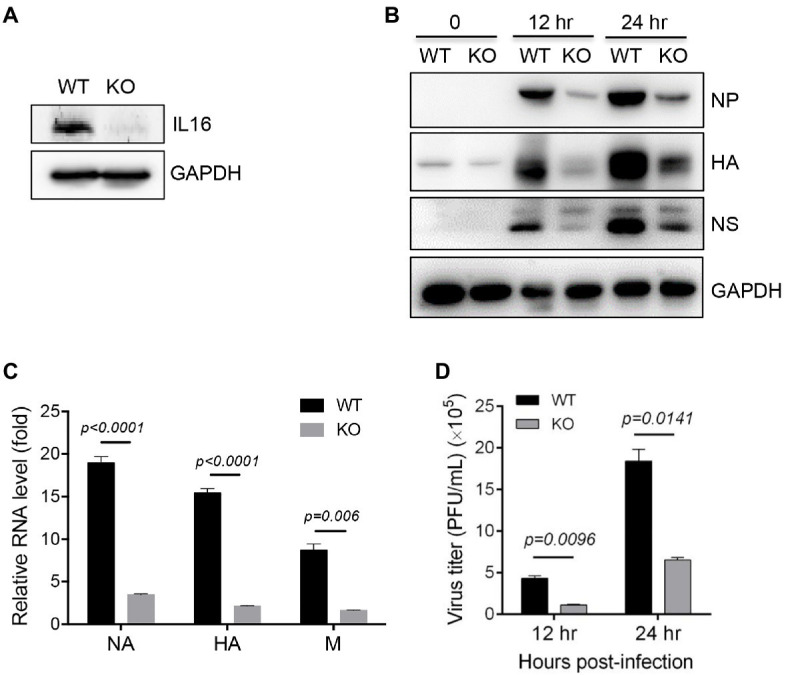
IL16 deficiency inhibits IAV replication. **(A)** Detection of IL16 expression in WT and IL16 KO mouse embryonic fibroblasts (MEFs) using immunoblot analyses, GAPDH was used as a loading control. **(B)** WT and IL16 KO MEFs were infected with PR8 virus (MOI=0.1) for 12 and 24h. The protein levels of NP, HA, and NS were detected by immunoblot analyses with the indicated antibodies. **(C)** WT and IL16 KO MEFs were infected with PR8 virus (MOI=0.1) for 24h. The relative mRNA levels of NA, HA, and M genes were determined by qRT-PCR. Histograms represent mean±SD of triplicate samples (two experiments). A value of *p*<0.05 was considered significant. **(D)** WT and IL16 KO MEFs were infected with PR8 virus (MOI=0.1) for 12 and 24h. The supernatant was collected and subjected to plaque assay for measuring virus titer. Histograms represent mean±SD of triplicate samples (three experiments). A value of *p*<0.05 was considered significant.

To further confirm IL16 function, WT and IL16 KO MEFs were prepared from WT and IL16 KO mice as previously described, respectively (Liu et al., 2020). The knockout efficiency was confirmed by immunoblot analyses (Figure 4A). WT and IL16 KO MEFs were infected with PR8 viruses at an MOI of 0.1 for 12h, and 24h, immunoblot analyses showed that IL16 deficiency significantly reduced the expression of NP, NS, and HA proteins of PR8 virus (Figure 4B). The mRNA levels of PR8 NA, HA, and M genes were also dramatically downregulated in IL16 KO MEFs as compared to the WT cells after PR8 infection for 24h (Figure 4C). Consistently, IL16 deficiency markedly decreased the production of new progeny PR8 viruses at 12 and 24h post-infection (Figure 4D). These results are indicative of the important role of IL16 in IAV infection.

### IL16 Blocks the Production of Type I IFN and ISGs

**Figure 5:**
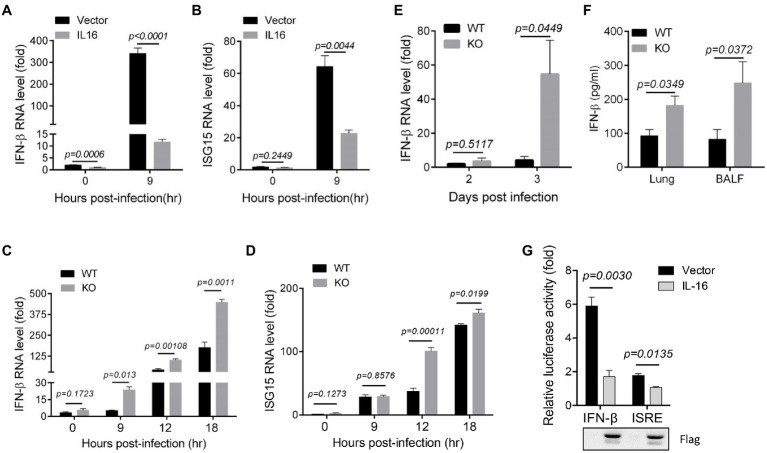
IL16 inhibits interferon (IFN)-β and IFN-stimulated genes (ISG) expression. **(A,B)** A549 cells were transfected with vector or IL16-expressing plasmid for 24h, followed by infection with PR8 virus (MOI=5) for the indicated time points. The infected cells were harvested and subjected to qRT-PCR analyses with the specific primers corresponding to IFN-β **(A)** and ISG15 **(B)**. **(C,D)** WT and IL16 KO MEFs were infected with PR8 virus (MOI=0.1) for the indicated time points, followed by qRT-PCR analyses with the primers corresponding to IFN-β **(C)** and ISG15 **(D)**. **(E,F)** C57BL/6 WT or IL16 KO mice (6–8weeks) were infected with mock (*n*=6) or PR8 virus (10,000 PFU/mouse, *n*=10). Lungs were harvested on day 2 and day 3 post-infection and detected for IFN-β through qRT-PCR analyses **(E)**, ELISA assay for IFN-β in the lung homogenates and BALF on day 3 post-infection **(F)**. **(G)** A549 cells were transfected with IFN-β or ISRE luciferase reporter plasmids, together with vector or IL16-Flag-expressing plasmid for 24h, followed by luciferase assay. IL16 expression was detected by immunoblot analyses with Flag antibody. Histograms represent mean±SD of triplicate samples (three experiments). A value of *p*<0.05 was considered significant.

Type I IFN and ISGs are critical for the host resistance to IAV infection and play important roles in inhibiting IAV replication (Garcia-Sastre et al., 1998; Koerner et al., 2007; Shim et al., 2017), we next examined whether IL16 affects the production of type I IFN. To test this, A549 cells were transfected with vector or IL16-expressing plasmids, followed by the infection with PR8 virus at an MOI of 5. The expression of IFN-β and ISG15 were detected at the indicated time points after infection. IL16 overexpression significantly reduced the level of IFN-β mRNA before and after PR8 infection (Figure 5A). ISG15 expression was also decreased in IL16-expressing cells after PR8 infection (Figure 5B), suggesting that IL16 overexpression represses type I IFN-β and ISG expression.

Next, we infected WT and IL16 KO MEFs with the PR8 virus at an MOI of 0.1. The qRT-PCR analyses showed that IL16 deficiency slightly increased IFN-β and ISG15 expression before PR8 infection, however, dramatically elevated IFN-β and ISG15 expression after PR8 infection (Figures 5C,D). Furthermore, we infected WT and IL16 KO mice with a sublethal dose (8,000 PFU) of PR8 viruses and detected the IFN-β mRNA level in lung homogenates on day 2 and day 3 post-infection. The IFN-β mRNA level in the lungs was significantly higher in the IL16 KO mice as compared with WT mice at day 3 post-infection (Figure 5E). In consistence, the IFN-β protein level was also higher in the lung homogenates and BALF samples of IL16 KO mice on day 3 post-infection as compared with WT mice (Figure 5F). Collectively, these data indicate that IL16 deficiency is reversely correlated with IAV-induced IFN-β and ISG15 expression.

To further assess whether IL16 can regulate ISRE and IFN-β activation, A549 cells were transfected with IFN-β or ISRE luciferase plasmid, together with IL16-expressing plasmid or vector alone. IL16 expression markedly blocked IFN-β and ISRE activation (Figure 5G). These data illustrate that IL16 could directly inhibit type I IFN activation, negatively regulate type I IFN and ISG expression, which might contribute to the role of IL16 in promoting IAV infection.

### IL16 Deficiency Alleviates IAV Infection and Lung Injury

**Figure 6:**
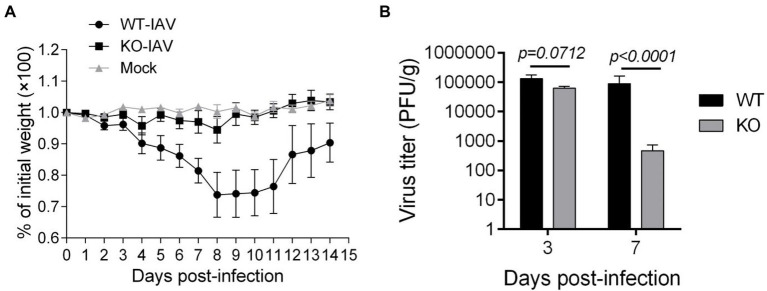
IL16 deficiency reduces the susceptibility to IAV infection in mice. Seven-week-old male WT and IL16 KO mice were intranasally infected with PR8 virus (8,000 PFU/mouse), and control mice received mock inoculation with PBS. **(A)** Daily weights of each mouse were measured within 14days after infection, 10 mice per group. **(B)** Lungs were collected from infected mice at day 3 and day 7 post-infection and pulmonary viral loads were measured by plaque assays using MDCK cells. Data shown are mean±SD, 10 mice per group. A value of *p* <0.05 was considered significant.

**Figure 7:**
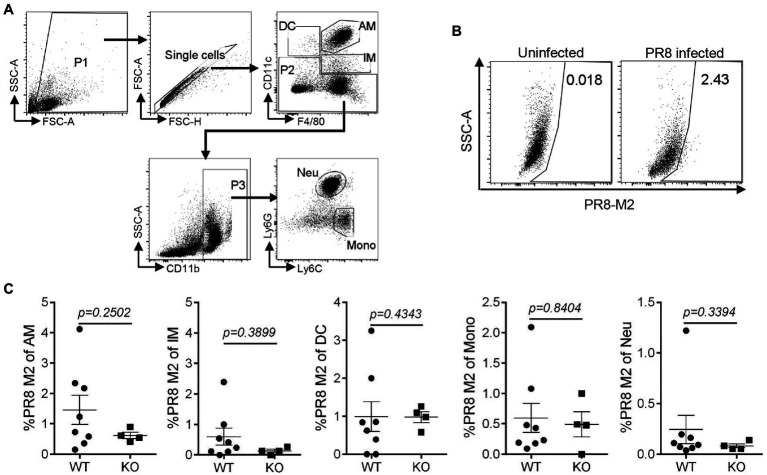
IL16 deficiency does not affect IAV infection in lung myeloid cell subsets. Seven-week-old male WT and IL16 KO mice were intranasally infected with PR8 virus (8,000 PFU), and control mice received mock inoculation with PBS. Lungs from the infected mice were harvested on day 2 post-infection. PR8 M2 expression in the myeloid cell subsets was analyzed using flow cytometry. **(A)** The gating strategy for myeloid cell subsets. AM, alveolar macrophages; IM, interstitial macrophages; DC, dendritic cells; Mono, monocytes; Neu, neutrophils. **(B)** Representative plot showing PR8 M2 expression in alveolar macrophages. **(C)** PR8 M2 expression in different myeloid cell subsets in the lungs of infected mice. The Data shown are representative of two independent experiments, eight mice for WT group and four mice for KO group. A value of *p*<0.05 was considered significant.

To assess the effect of IL16 on the mouse susceptibility to IAV infection, 7- to 8-week-old mice were intranasally infected with 8,000 PFU of PR8 viruses. The weight of each mouse was monitored after infection. As expected, PR8-infected WT mice displayed rapid loss of body weight and reached about 25–30% in the weight loss by days 8–10 post-infection, whereas IL16 KO mice maintained normal body weight, similar to the mock-infected WT mice (Figure 6A). Next, we examined the viral load in the lung homogenates of the infected mice at day 7 post-infection. Plaque assays revealed a significant reduction of PR8 virus titers in the lungs of IL16 KO mice as compared to WT mice (Figure 6B), suggesting that IL16 deficiency attenuates IAV infection and protects the weight loss of the infected mice. IL16 has been reported to involve in the HIV replication in macrophages, dendritic cells, and monocytes (Gao et al., 1997; Maciaszek et al., 1997; Amiel et al., 1999), we next detected PR8 M2 expression in alveolar macrophages (AM), interstitial macrophages (IM), dendritic cells (DC), monocytes (Mono), and neutrophils (Neu) in the lungs of WT and IL16 KO mice at day 2 post-infection. A low level of PR8 M2 expression was observed in the myeloid cell subsets and there was no significant difference between WT and KO mice (Figure 7).

**Figure 8:**
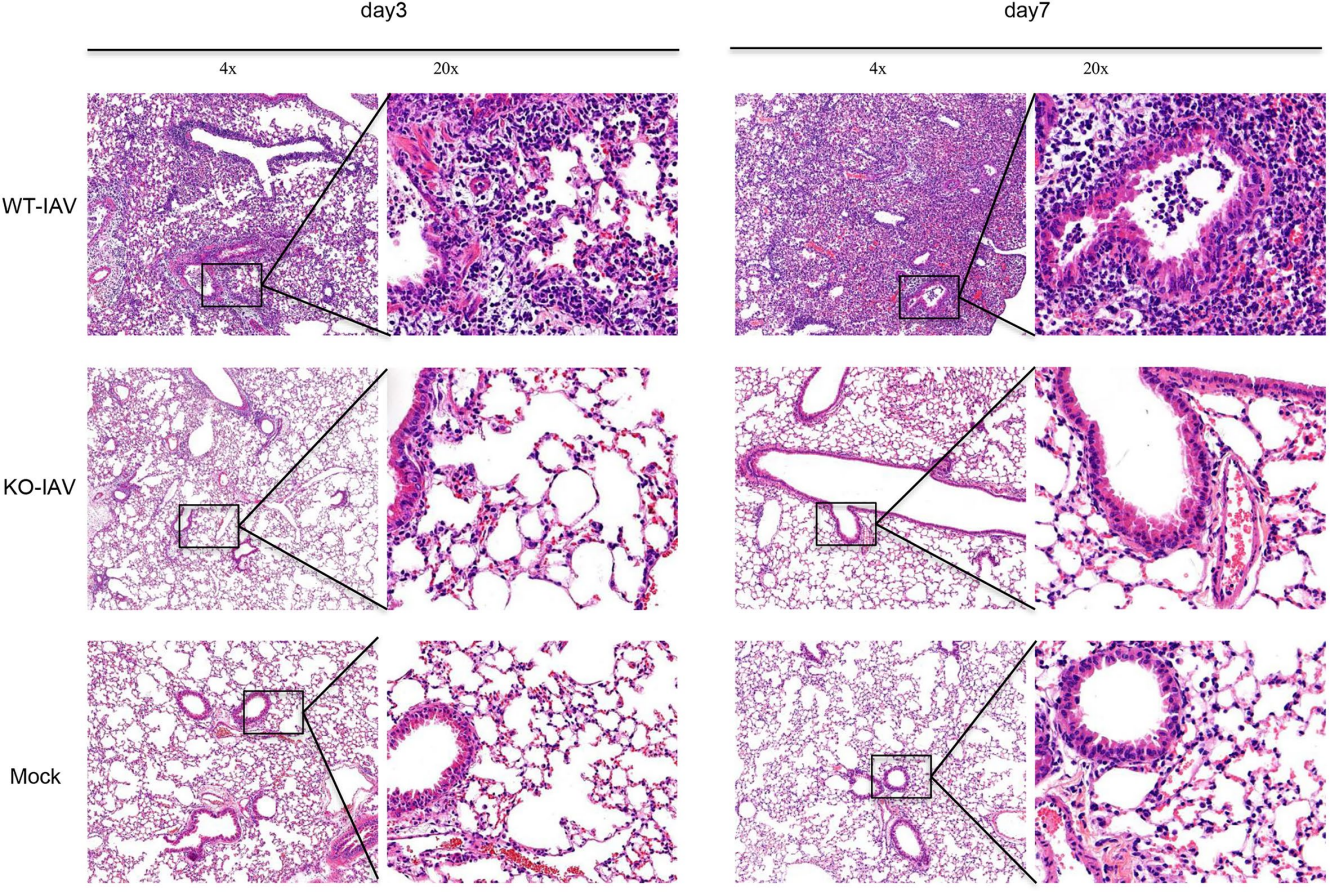
IL16 deficiency alleviates lung injury after IAV infection. Seven-week-old male WT and IL16 KO mice were intranasally infected with PR8 virus (8,000 PFU), and control mice received mock inoculation with PBS. Lungs from each infected mouse were isolated on day 3 and day 7 post-infection and fixed for subsequent H&E staining. Pulmonary histological images were taken using a microscope at resolutions of 4×, 20× magnification. Data shown are representative of two independent experiments; *n*=5 per group per experiment.

Influenza A virus infection can lead to lung injury and cause a histopathological change in the lung (Fukushi et al., 2011; Herold et al., 2015). We performed a histologic examination to analyze the lung injury for the infected mice at day 3 and day 7 post-infection. The H&E staining revealed alveolar damage and infiltrating immune cells in the lungs of PR8-infected WT mice, whereas age-matched PR8-infected IL16 KO mice and PBS mock-infected mice showed the normal lung histology (Figure 8), suggesting that IL16 deficiency alleviates IAV-induced lung injury, in line with the lower viral load and less pronounced body weight loss as observed above.

## Discussion

Influenza viruses replicate within the nucleus of the host cells, utilizing a combination of viral and cellular mechanisms to complete their successful replication (Shim et al., 2017; Dou et al., 2018). Meanwhile, the host immune system has evolved various strategies to limit replication. Multiple cellular proteins have been characterized to be essential or important for IAV replication by mediating virus entry, fusion, uncoating, viral RNA transcription, vRNP transport, and the release of new virions (Shim et al., 2017). Our current study demonstrates that IL16 promotes the host susceptibility to IAV infection by acting as a supporting factor.

Given that CD4 acts as a receptor for IL16 in T cells as reported previously (Cruikshank et al., 1994), the function of IL16 on infectious diseases has mainly been investigated in HIV-1 infection. The recombinant C-terminal 130-amino acid portion of IL16 is shown to inhibit HIV-1 mRNA expression and suppress HIV-1 replication (Baier et al., 1995; Zhou et al., 1997; Amiel et al., 1999). When IL16 was added before HIV-1 infection, it could repress HIV-1 LTR expression in lymphoid but not monocytoid cells (Maciaszek et al., 1997). But when added during or early after infection, IL16 could repress HIV-1 entry and replication in macrophages and dendritic cells (Truong et al., 1999). IL16 does not suppress HIV-1 replication in naturally infected peripheral blood mononuclear cells (Gao et al., 1997), suggesting that IL16 function in HIV infection is cell type-specific. Our data reveal that IL16 has little effect on IAV infection in myeloid cell subsets of the infected lungs, which might not contribute to the role of IL16 in promoting IAV replication. Although CD4 is thought to be the primary receptor for IL16 as supported by indirect evidence, the direct interaction between IL16 and CD4 has not been shown so far. Other studies have identified CD9 as a potential alternative receptor for IL16 in mast cells and epithelial cells (Mathy et al., 2000; Qi et al., 2006; Blake et al., 2018), indicating that IL16 might use different receptors in different cell types. Our recent study has shown that IL16 does not affect gammaherpesvirus lytic replication, but inhibits lytic reactivation from latency through regulation of STAT3-p21 axis, acting as a positive regulator for gammaherpesvirus latency (Liu et al., 2020). The diverse functions of IL16 illustrate that IL16 is a multifunctional cytokine. The underlying mechanism of IL16 regulation of IAV replication is not well resolved yet, partly might be through inhibiting type I IFN and ISG expression.

Interferon and ISGs are important to limit viral infection including IAV infection. Lung epithelial cells and macrophage are main sources of type I IFN upon IAV infection (Jewell et al., 2007). Our data show that IL16 can directly inhibit type I IFN activation, which might lead to the inhibition of type I IFN and ISG production, consequently supporting IAV infection. Due to lack of IL16/type I IFN double KO MEFs, type I IFN receptor KO MEFs, or effective IFN-β blocking antibody, we could not make a direct conclusion that IL16 suppression of type I IFN signaling is the underlying mechanism for its role in promoting IAV infection, which needs to be further investigated.

Interleukin 16 is expressed by a wide range of cell types, including immune cells and non-immune cells (e.g., fibroblasts, epithelial cells; Yadav et al., 2010). Human lung epithelial A549 cells express IL16 as evidenced by RT-PCR and ELISA detection (Cheng et al., 2001). We also detected endogenous expression of IL16 A549 cells by immunoblot analyses. Although A549 cells do not express CD4, they are known to express CD9 receptor on the cell surface (Blake et al., 2018). Furthermore, our data show that IL16 regulation of gammaherpesvirus reactivation is independent of its secreted form, not through the receptor-mediated mechanism (Liu et al., 2020). We do not rule out that IL16 promotion of IAV infection is likely in a receptor-independent manner, in agreement with the previous reports that some unknown non-CD4-based mechanisms are occurring in IL16-responding cells (Yoshida et al., 2001; Yadav et al., 2010).

It is worth noting that although the PR8 virus titer is significantly lower in IL16 KO MEFs as compared to WT MEFs, PR8 virus production appears to show similar fold increases between WT and KO cells from 12 to 24h post-infection, raising the possibility that the initial infection rate is different between WT and KO cells, or KO MEFs exhibit a higher level of PR8-induced IFN-β and ISG expression as compared to the WT cells, and show the similar increase rate of IFN-β expression to WT cells from 0 to 9, 12, and 18h after PR8 infection as shown in Figure 5, which, in turn, correspondingly suppresses IAV replication and account for the reduction of PR8 virus production in IL16 KO cells.

We have recently revealed that IL16 deficiency promotes Th1 and CTL response against IAV infection (Jia et al., 2020). Importantly, IFN-α/β signaling is known to promote Th1 responses (Nguyen et al., 2002; Le Bon and Tough, 2008; Longhi et al., 2009), and has a direct role in linking innate and adaptive responses by providing the “third signal” needed by naive CD8+ T cells responding to pathogen and costimulatory ligands (Curtsinger et al., 2005). It is reasonable to assume that promotion of Th1 and CTL responses in IL16-deficient mice might be attributed to the strengthening of type I IFN production caused by IL16 deficiency, highlighting that both type I IFN and the host cellular immunity mediated by IL16 deficiency possibly contribute to the protection of IAV-infected mice from the weight loss and lung injury. In addition, natural killer (NK) cells, which confer similar cytotoxic effect as CTLs but have distinct approaches to control their activity and specificity (Chiang et al., 2013), might also be influenced by IL16, since type I IFNs act directly on NK cells to promote their activation, proliferation, and cytotoxic function during viral infection (Stackaruk et al., 2013; Madera et al., 2016). Whether the function of NK cells is affected by IL16 deserves further confirmation. The effect of IL16 on various immune cells might be multifaceted and a more detailed mechanism needs to be illustrated in the future.

Although we have clinical data showing that the serum level of IL16 is elevated in IAV-infected children, we still could not make a decisive conclusion about whether IL16 mediates IAV infection and symptom severity clinically, which needs more clinical analyses. At least, our *in vitro* data and mouse infection model provide the insight that IL16 displays a comprehensive functional activity in response to viral infection and prospect IL16 as a potential therapeutic target.

## Data Availability Statement

The original contributions presented in the study are included in the article/supplementary material, further inquiries can be directed to the corresponding authors.

## Ethics Statement

The studies involving human participants were reviewed and approved by Ethics Committee of the Children’s Hospital of Fudan University. Written informed consent to participate in this study was provided by the participants’ legal guardian/next of kin. The animal study was reviewed and approved by Animal Ethics Committee of Institut Pasteur of Shanghai.

## Author Contributions

RJ, CJ, LL, CH, JX, and XL conceived and designed the experiments. RJ, CJ, LL, CH, LL, and MX performed the experiments. RJ and LL analyzed the data. RJ and XL wrote the paper. All authors contributed to the article and approved the submitted version.

## Funding

This work was supported by the National Key R&D Program of China (2016YFA0502100).

## Conflict of Interest

The authors declare that the research was conducted in the absence of any commercial or financial relationships that could be construed as a potential conflict of interest.

## Publisher’s Note

All claims expressed in this article are solely those of the authors and do not necessarily represent those of their affiliated organizations, or those of the publisher, the editors and the reviewers. Any product that may be evaluated in this article, or claim that may be made by its manufacturer, is not guaranteed or endorsed by the publisher.
